# Purification of Maize Nucleotide Pyrophosphatase/Phosphodiesterase Casts Doubt on the Existence of Zeatin *Cis*–*Trans* Isomerase in Plants

**DOI:** 10.3389/fpls.2017.01473

**Published:** 2017-08-23

**Authors:** Tomáš Hluska, Marek Šebela, René Lenobel, Ivo Frébort, Petr Galuszka

**Affiliations:** ^1^Department of Molecular Biology, Centre of the Region Haná for Biotechnological and Agricultural Research, Faculty of Science, Palacký University Olomouc Olomouc, Czechia; ^2^Department of Protein Biochemistry and Proteomics, Centre of the Region Haná for Biotechnological and Agricultural Research, Faculty of Science, Palacký University Olomouc Olomouc, Czechia

**Keywords:** zeatin, isomerization, maize, nucleotide pyrophosphatase/phosphodiesterase, flavins

## Abstract

Almost 25 years ago, an enzyme named zeatin *cis–trans* isomerase from common bean has been described by Bassil et al. (1993). The partially purified enzyme required an external addition of FAD and dithiothreitol for the conversion of *cis*-zeatin to its *trans-* isomer that occurred only under light. Although an existence of this important enzyme involved in the metabolism of plant hormones cytokinins was generally accepted by plant biologists, the corresponding protein and encoding gene have not been identified to date. Based on the original paper, we purified and identified an enzyme from maize, which shows the described zeatin *cis–trans* isomerase activity. The enzyme belongs to nucleotide pyrophosphatase/phosphodiesterase family, which is well characterized in mammals, but less known in plants. Further experiments with the recombinant maize enzyme obtained from yeast expression system showed that rather than the catalytic activity of the enzyme itself, a non-enzymatic flavin induced photoisomerization is responsible for the observed zeatin *cis–trans* interconversion *in vitro*. An overexpression of the maize nucleotide pyrophosphatase/phosphodiesterase gene led to decreased FAD and increased FMN and riboflavin contents in transgenic *Arabidopsis* plants. However, neither contents nor the ratio of zeatin isomers was altered suggesting that the enzyme is unlikely to catalyze the interconversion of zeatin isomers *in vivo*. Using enhanced expression of a homologous gene, functional nucleotide pyrophosphatase/phosphodiesterase was also identified in rice.

**GRAPHICAL ABSTRACT:**
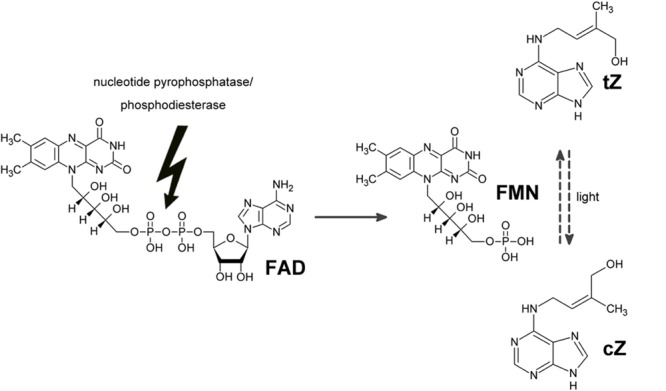
Proposed mode of action of “zeatin *cis-trans* isomerase”. Instead of catalyzing the isomerization using FAD and light as cofactors, the enzyme hydrolyses FAD and formed FMN is more efficient non-enzymatic catalyst of the isomerization. Thus the apparent increase in zeatin isomerization rate upon enzyme addition was observed.

## Introduction

*N*^6^-(4-hydroxy-3-methyl-but-2-enyl)adenine, known as zeatin due to its discovery in maize (*Zea mays*), belongs to an important class of plant hormones called cytokinins that regulate many physiological processes (Werner and Schmülling, 2009). Zeatin is found throughout the plant kingdom in the form of two geometrical isomers denoted as *cis*- and *trans*-zeatin, respectively. *trans-*Zeatin biosynthesis occurs either directly from adenylate and a hydroxylated side chain precursor *(E)-*4-hydroxy-3-methyl-but-2-enyl diphosphate) by adenylate dimethylallyltransferase (EC 2.5.1.27; Kakimoto, 2001) or by further hydroxylation of another cytokinin isopentenyladenine [(γ,γ-dimethylallylamino)purine] by specific cytochrome P450 (Takei et al., 2004). On the other hand, the only route of the formation of *cis*-zeatin is the hydrolysis of tRNAs that had been previously prenylated on *N*^6^-amino group of certain adenosine phosphate residues by tRNA dimethylallyltransferase (EC 2.5.1.75), which was evidenced by the decrease in *cis-*zeatin levels in *trna:ipt* knock-out mutants of *Arabidopsis* (Miyawaki et al., 2006) and *Physcomitrella* (Lindner et al., 2014).

Since *cis-*zeatin is the predominant cytokinin in many plant species (Gajdošová et al., 2011), there are still alternative pathways under consideration. The expression of tRNA dimethylallyltransferase genes is rather uniform throughout the plant ontogenesis, not responding to stimuli or any changes in cytokinin content. Nevertheless, an involvement of development- and/or stimuli-dependent RNase(s) liberating *cis*-zeatin cannot be ruled out. In addition, there is still a need for specific hydroxylase forming the *cis-*hydroxylated side-chain. Such a *cis*-hydroxylase encoded by *miaE* gene was found in the bacterium *Salmonella typhimurium* (Persson and Björk, 1993), but no homologue has been identified up to now in plants.

Therefore it was generally accepted that *cis*-zeatin could be produced from its *trans-* isomer by zeatin *cis–trans* isomerase, an enzyme partly purified and reported almost 25 years ago (Bassil et al., 1993). Nevertheless, the existence of such an enzyme has been disputed, as it was never identified; e.g., upon feeding plants with a radioactive cytokinin, no isomerization was observed in maize (Yonekura-Sakakibara et al., 2004). The only exception was 5–9% of radioactivity recovered as *trans-*zeatin upon feeding potato tubers with *cis-*zeatin (Suttle and Banowetz, 2000). However, the radioactive cytokinin was applied in the dark, while the reaction catalyzed by the enzyme *in vitro* was described to require light. Furthermore, *Arabidopsis* multiple knock-out plants in adenylate dimethylallyltransferase genes show decreased levels of *trans-*zeatin and isopentenyladenine but not *cis*-zeatin, while tRNA dimethylallyltransferase gene knock-out specifically reduces *cis-*zeatin (Miyawaki et al., 2006). Neither of these results would have been plausible, if significant *cis–trans* isomerization of zeatin had occurred *in planta*.

Here we report on the purification of the protein responsible for the *in vitro* zeatin *cis–trans* isomerase activity described earlier. The protein was purified from maize and identified by mass spectrometry as a nucleotide pyrophosphatase/phosphodiesterase. A recombinant enzyme was produced using yeast *Pichia pastoris* expression system and its substrate specificity determined. To assess a possible role of nucleotide pyrophosphatase/phosphodiesterase in cytokinin metabolism, experimental plants with an altered expression of the encoding gene were prepared and characterized.

## Materials and Methods

### Purification of the Protein with Zeatin *Cis–Trans* Isomerase Activity

Whole maize cobs with immature seeds (930 g), approximately 10 days after pollination, were collected in the field and homogenized using a food processor with 1 L of 50 mM Tris/HCl, pH 8.0, at 4°C for 1 h. After a centrifugation at 4,800*g*, the pellet was re-extracted with the same volume of fresh buffer and centrifuged again. Ballast proteins were precipitated with additions of protamine sulfate (1 g per 10 g of proteins), manganese(II) chloride (final concentration 7.5 mM) and ammonium sulfate (20% saturation, 114 g L^-1^) according to published protocol (Šebela et al., 2000). After the addition of each precipitant, the solution was stirred for 10 min and then centrifuged at 4,800 *g* for 10 min. The supernatant was loaded onto a DEAE-Sepharose (GE Life Sciences) 5.0 cm i.d. × 30 cm column equilibrated with 50 mM Tris/HCl, pH 8.0, containing ammonium sulfate at 20% saturation. The flow-through fraction was desalted using a MiniKros^®^ hollow fiber tangential flow filtration system with a 10-kDa polysulfone module (Spectrum Laboratories) and loaded on a High Q (Bio-Rad) 1.5 cm i.d. × 20 cm column equilibrated with 50 mM Tris/HCl, pH 8.0. The bound proteins were eluted with a gradient of KCl from 0 to 1.0 M. Fractions showing the zeatin *cis–trans* isomerase activity were pooled and transferred to 20 mM Tris/HCl buffer, pH 7.4, containing 0.5 M NaCl and 5 mM of each MgCl_2_, MnCl_2_, and CaCl_2_ using Amicon^®^ Ultra centrifugal filters (10,000 NMWL; Merck Millipore). The sample was then loaded on a Concanavalin A-Sepharose (Sigma–Aldrich) 1.0 cm i.d. × 10 cm column equilibrated with the same buffer and eluted with the buffer containing 1 M methyl α-D-mannopyranoside. The sample was then transferred to 50 mM Tris/HCl, pH 8.0 using the above centrifugal filters.

The enzyme was further purified using an FPLC BioLogic DuoFlow 10 system (Bio-Rad). The sample was first loaded onto a Resource Q 6 mL column (GE Life Sciences) equilibrated with 50 mM Tris/HCl, pH 8.0, and eluted with a linear gradient up to 0.5 M KCl. Active fractions were pooled, transferred to 5 mM K-phosphate, pH 7.0, containing 0.1 M NaCl and 0.5 mM CaCl_2_. The recovered proteins were loaded onto a Bio-Scale CHT5-I column (Bio-Rad) and eluted with 0.75 M K-phosphate buffer, pH 7.0. Active fractions were pooled and transferred to 50 mM Tris/HCl, pH 8.0. Concentrated enzyme solution was loaded onto a HiTrap Blue HP column (GE Life Sciences) and eluted with 2 M KCl.

Finally, the purified enzyme was applied to Novex^®^ isoelectric focusing electrophoresis gels (ThermoFisher Scientific) with pH 3-10 buffers. The gels were run at a constant voltage of 100 V for 1 h, then at 200 V for 1 h and finally at 400 V for an additional hour. The margin lines were excised and silver-stained. Protein bands were excised from the rest of the gel slab in accordance to the staining and crushed by pressing through a syringe several times. Afterward it was extracted overnight with 100 μL of McIlvaine buffer pH 7.5 at 4°C.

### Protein Identification by Mass Spectrometry

After SDS-PAGE followed by Coomassie staining, protein bands of interest were excised from the gel slab and processed for in-gel digestion by a modified trypsin (Šebela et al., 2006). Peptides from the digests were desalted using ZipTip C18 pipette tips (Merck Millipore Ltd.) according to manufacturer’s instructions and finally reconstituted in 10 μl of 0.1% (v/v) trifluoroacetic acid (TFA). Prior to MALDI mass spectrometry (MS) and tandem mass spectrometry (MS/MS), α-cyano-4-hydroxycinnamic acid (a matrix compound) was dissolved to 0.7 mg ml^-1^ in a solvent mixture containing 85% (v/v) acetonitrile, 15% (v/v) water, 0.1% (v/v) TFA and 1mM NH_4_H_2_PO_4_. An aliquot (0.5 μl) of the sample solution corresponding to an initial protein amount of around 200 ng was spotted onto the target (MTP AnchorChipTM 384 BC; Bruker Daltonik), immediately mixed with 0.5 μl of the matrix solution and left to dry at laboratory temperature.

MS and MS/MS analyses were performed on an ultrafleXtreme MALDI-TOF-TOF instrument equipped with a LIFT cell and Smartbeam-II laser operating at a repetition rate up to 2 kHz (Bruker Daltonik). All mass spectra were obtained in the reflectron positive ion mode. The mass spectrometer was controlled by flexControl 3.3 software for acquisition and flexAnalysis 3.3 for spectra processing. The accelerating voltages in the ion source for MS and MS/MS analyses were 25 and 7.5 kV, respectively. In MS/MS mode (no collision gas was used), an accelerating potential of 19 kV was applied to fragments (coming from a timed ion gate) in the LIFT cell. The instrument was calibrated externally using peptide standards supplied by the manufacturer. Manual mass spectra acquisitions were done from 2,000 laser shots in the MS mode and 5,000–10,000 shots in the MS/MS mode. The following settings were applied for MS/MS in an AutoXecute method: primary choice mass range of precursors: 750–3000; number of precursor masses: 15; peak intensity: >800; peak quality factor: >30; signal/noise: >7; FAST minimal fragment mass: 250; LIFT: measure fragments only.

Combined MS/MS datasets were processed by flexAnalysis 3.3, uploaded to ProteinScape 3.0 (Bruker Daltonik) and searched against the NCBI non-redundant database with the Mascot 2.2 search engine (Matrix Science, London, United Kingdom). The search parameters were as follows: Viridiplantae (green plants) were set as a taxonomy; trypsin was set as a protease with 1 missed cleavage allowed; carbamidomethylation of cysteine was set as a fixed modification and methionine oxidation as a variable modification; +1 was set as a peptide charge; monoisotopic masses were considered; other settings: instrument – MALDI-TOF-TOF, significance threshold – *p* < 0.05, peptide mass tolerance – 25 ppm, MS/MS fragment mass tolerance – 0.7 Da.

### Design and Synthesis of *ZmNPP* Gene

A maize nucleotide pyrophosphatase/phosphodiesterase (*ZmNPP*) gene was synthesized by GeneArt service of ThermoFisher Scientific. Minor alterations from the native sequence were implemented as follows: a CAT triplet was placed before the start codon to form an *Nde*I restriction site, the sequence stretch GCCTCC from the position 107 was changed to GCTAGC to form an *Nhe*I restriction site, which allows to clone *ZmNPP* fragment without predicted signal sequence, and the triplet CTT at the position 901 was changed to TTG to remove a *Hind*III restriction site. Neither of these changes led to an amino acid substitution. The TGA stop codon was changed to TAA to form a new *Hind*III restriction site and an adjacent *Xho*I site was added to yield a sequence TAAGCTTCTCGAG. All changes were made in order to clone the gene into plasmids for expression in *E. coli.* Gateway *att*B1 and *att*B2 sequences were added up- and down-stream of the coding sequence, respectively.

### Preparation of Recombinant ZmNPP from *Pichia pastoris* Expression System

The *ZmNPP* gene was amplified from synthesized DNA with the following primers: P1_fw (5′-G*GAATTC*ATGGCGTCTCCGCCCCACTC-3′) and P2_rev (5′-GCCG*CTCGAG*TTTTGTTCGGCAACAGAATCGTGCC-3′) with *Eco*RI and *Xho*I restriction sites, respectively, shown underlined in italics. After restrictions with the respective endonucleases, the gene was cloned into a vector pPICZα (ThermoFisher Scientific). The plasmid was then linearized with *Mss*I. Chemically induced transformation to *Pichia pastoris* X-33 was performed in accordance with the manufacturer’s protocol.

Transformed yeasts were cultivated in a bioreactor Biostat^®^ Cplus (Sartorius). The vessel was filled with water up to 8.5 L containing 134 g of yeast nitrogen base without amino acids (ThermoFisher Scientific), 400 mL of glycerol and 2 mL of antifoam A (Sigma–Aldrich) and steam sterilized. After cooling down, 1 L of 1 M potassium phosphate, pH 7.0, and 50 mL of filter-sterilized PTM_1_ Trace Salts (Pichia Fermentation Process Guidelines, ThermoFisher Scientific) were added and the conditions were set as follows: agitation 400 rpm, air flow 2 vvm, temperature 28°C, and pH 7.0. After 3 h, 0.5 L of an overnight preculture of *Pichia pastoris* harboring pPICZα::ZmNPP was added and agitation was set to keep the concentration of dissolved oxygen above 20% of saturation. After 24 h of fermentation, 50% glycerol containing 5% PTM_1_ Trace Salts was fed continuously to the bioreactor at the rate of 2 mL min^-1^. After another 24 h, an induction of protein expression with methanol started, increasing the rate up to 3 mL min^-1^, while the feeding with glycerol was decreased linearly. The methanol was fed to the bioreactor for 3 more days.

Yeast cells were then collected by a centrifugation at 4,800*g* for 30 min. The medium was conditioned with ^1^/_40_ volume of 1 M Tris and 2 M NaCl, run through a DEAE-Sepharose 5.0 cm i.d. × 30 cm column, concentrated and conditioned to 20 mM Tris/HCl, pH 8.0, on a SartoJet pump with Sartocon^®^ Slice Ultrasart polyethersulfone 10-kDa NMWCO cassettes (Sartorius). The enzyme was further purified on the High Q 1.5 cm i.d. × 20 cm and HiTrap Blue HP columns as described above.

### Enzyme Activity Assays

The interconversion of zeatin isomers was measured with 0.2 mM *cis-*zeatin in 100 mM McIlvaine buffer containing 20 mM MgCl_2_, 0.1 mM FAD and 0.8 to 2.0 mM dithiothreitol. Upon mixing, the samples were incubated at 37°C under white fluorescent light (500 μE m^-2^ s^-1^). The reaction was stopped by the addition of two volumes of methanol after 1 h. For each assay, a control reaction was set-up, where the enzyme sample was boiled for 5 min prior to addition of the other components and the enzymatic activity was calculated from the difference in the concentration of *trans*-zeatin between the two reactions. Zeatin content was analyzed on a Nexera UFLC (Shimadzu) equipped with a Zorbax RRHD Eclipse Plus C18 column, 2.1 mm i.d. × 50 mm, 1.8 μm (Agilent) thermostated at 40°C. Zeatin isomers were eluted with 15 mM formic acid, set up with ammonium hydroxide to pH 4.0 at the flow rate of 0.4 mL min^-1^ with gradient of methanol as follows (min/%): 0.0/22; 3.0/22; 4.0/90; 5.5/90; 6.0/22; 8.0/22 and their content determined from peak areas at 268 nm using standard compounds. A typical chromatogram of the reaction is shown in Supplementary Figure S1A.

The assay of nucleotide pyrophosphatase/phosphodiesterase activity was done in identical manner, but without *cis*-zeatin and the reaction mixture was kept in dark. Moreover, dithiothreitol was omitted in the assays with purified recombinant protein as no longer needed. The samples were analyzed on the same UFLC system and conditions as above, but eluted with 20 mM phosphoric acid (set up to pH 6.5 with ammonium hydroxide) using the following methanol gradient (min/%): 0.0/15; 1.0/15; 4.0/22; 4.5/25; 5.0/95; 7.0/95; 7.5/15; 10.0/15 and monitored at 449 nm. A typical chromatogram of the reaction is shown in Supplementary Figure S1B. In plant samples, extracted flavins were analyzed by the same procedure but quantified on a FP-2020 Plus fluorescence detector (JASCO) with excitation at 265 nm and emission at 530 nm using standard compounds.

To determine the nucleotide pyrophosphatase/ phosphodiesterase activity with various substrates, the same reaction was set up with 0.5 mM substrate and with an appropriate amount of the purified recombinant enzyme hydrolyzing maximally 40% of the substrate. The reaction was then stopped by pipetting aliquots at time-points 0, 10, 20, and 30 min into two volumes of methanol. The samples were all centrifuged at 21,000 *g* for 10 min and mixed with 15 mM triethylamine set with phosphoric acid to pH 6.0 in such a ratio to bring methanol to 5% and filtered using 0.22 μm Costar^®^ Spin-X^®^ centrifuge tube filters (Corning Inc.) at 10,000*g* for 3 min. The samples were then analyzed by the UFLC equipped with a Polaris 180Å C18-A, 2.0 mm i.d. × 150 mm, 3 μm (Agilent) thermostated at 35°C using a methanol gradient in 15 mM triethylamine (set up to pH 6.0 with phosphoric acid) at the flow rate of 0.2 mL min^-1^. The gradient was set up as follows (min/%): 0.0/10; 1.5/10; 4.0/40; 4.5/60; 7.0/60; 7.5/10; 10.0/10. The analytes were quantified using UV-Vis detector using standard compounds.

To determine enzyme specific activity, protein concentration was estimated using a Bio-Rad Protein Assay Dye Reagent Concentrate (Bio-Rad) with bovine serum albumin as a calibration standard with linearization (Ernst and Zor, 1996).

### Expression of the *ZmNPP* Gene in Tomato Hairy Roots

The gene was cloned into pGWB17 vectors (35S promoter, kanamycin and hygromycin resistance) using Gateway protocol (ThermoFisher Scientific) and electroporated into *Agrobacterium rhizogenes* strain 15834, which was used to transform tomato as described previously (Šmehilová et al., 2009). The main root was used for propagation; 2-cm long tips of lateral roots were used for the determination of gene expression and activity level and the rest was used for genotyping. Gene expression was determined as described previously (Šmehilová et al., 2009) with primers ZmNPP_fw (5′-CCCCAACCACTACTCCATCGT-3′) and ZmNPP_rev (5′-TCGTGGTTTTTCATGGTGAAGT-3′).

### Expression of the *ZmNPP* gene in *Arabidopsis*

*Arabidopsis* plants expressing maize nucleotide pyrophosphatase/phosphodiesterase gene cloned into the pGWB17 vector were prepared from *Arabidopsis thaliana* ecotype Col-0 using a floral-dip method (Clough and Bent, 1998). Homozygous plants were selected by PCR detection of the inserted *ZmNPP* gene. A plant was considered homozygous when at least 100 offspring plants carried the insert and at the same time there was no negative offspring plant. The plants were grown for 4 weeks in an environmental chamber (16 h fluorescence light intensity of 150 μE m^-2^ s^-1^/8 h dark, 22°C, 55% relative humidity) in the soil. Third to sixth leaves were collected and pooled from 4 plants into each sample.

### Cultivation of Rice Plants with Enhanced Expression of Nucleotide Pyrophosphatase/Phosphodiesterase

Rice (*Oryza sativa japonica*) T-DNA insertional line PFG_2B-60145.L with the corresponding wild type Hwayoung were purchased from POSTECH, South Korea. The line has an insertion in the promoter sequence of the gene LOC_Os01g10020, 300 bp upstream of the start codon, which contains the promoter sequence of *OsTubA1* gene. Rice seeds were germinated in Agroperlit (Perlit); after 2–3 weeks the seedlings were planted into the soil and grown for 2 weeks in an environmental chamber (12 h fluorescence light intensity of 250 μE m^-2^ s^-1^, 28°C/12 h dark, 25°C, 65% relative humidity). Then shoots were collected from each plant separately with the exception of the albino plants that were pooled from two plants into one sample.

### Analysis of Transgenic Plants

Enzymatic activity and protein content were assayed as above using samples prepared from 0.1 g of the leaves that were crushed in liquid nitrogen and extracted with 0.2 mL of 50 mM Tris/HCl, pH 8.0. Total flavins were first extracted with methanol/methylene chloride (9:10) from 5 mg of starting material as described previously (Hiltunen et al., 2012) then the sample was extracted again with 10 mM sodium phosphate containing 10% (v/v) acetonitrile. The whole procedure was conducted in a dim light. Finally, 10 μL of a mixture of both extracts (1:1) was mixed with 50 μL of 20 mM ammonium phosphate, pH 6.5, and analyzed using UFLC with fluorescence detector as above on the same type of column, but 150 mm long. Chlorophyll content was determined using published method (Porra et al., 1989). Cytokinins in *Arabidopsis* plants were determined using published method (Svačinová et al., 2012).

## Results

### Purification of the Protein with Zeatin *Cis–Trans* Isomerase Activity

**FIGURE 1:**
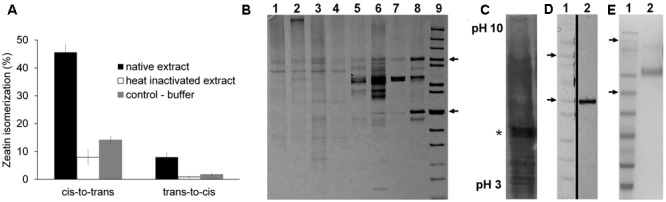
Purification and identification of the protein with zeatin *cis–trans* isomerase activity. **(A)** Typical conversion of zeatin isomers (0.2 mM) by maize kernel extracts after incubated in 100 mM McIlvaine buffer containing 20 mM MgCl_2_, 0.1 mM FAD and 2.0 mM dithiothreitol, at 37°C under white fluorescent light for 2 h; determined by UFLC; graph depicts average of three technical replicates with standard deviation. **(B)** SDS-PAGE of the individual protein purification steps: 1, crude extract; 2, extract after protein precipitation; 3, fraction from DEAE-Sepharose; 4, High Q; 5, Concanavalin A; 6 Resource Q; 7 Bio-Scale CHT5-I; 8 HiTrap Blue HP; 9, MW marker, 100 and 50 kDa indicated by arrows. **(C)** Isoelectric focusing gel with the sample after HiTrap Blue HP (silver-stained lane); the rest of the gel slab was sliced, proteins extracted and activity detected in the band indicated by the asterisk. **(D)** SDS-PAGE of the isolated active protein: 1, MW marker, 100 and 50 kDa indicated by arrows; 2, purified active protein; the vertical black line denotes combining of two image parts. **(E)** SDS-PAGE of the isolated recombinant ZmNPP protein: 1, MW marker; 2, purified enzyme from the yeast growth medium.

When we examined extracts from several plant species including maize, *Arabidopsis*, common bean, wheat and rice, the highest activity was found in maize, detectable in all developmental stages and organs (results not shown). Maize immature kernels were therefore chosen as the starting material for enzyme purification. As shown in **Figure 1A**, the rate of conversion of *cis-* to *trans*-zeatin was about 5-fold higher than the opposite reaction, both with a relatively high background after boiling that reached up to 25% of the rate with native extracts.

The whole purification procedure was quite tedious and required a series of six chromatographic steps to obtain 0.7 mg of a final enzyme preparation from almost one kilogram of maize cobs with kernels at the stage of liquid endosperm. The progress of individual purification steps with 10% activity yield resulted in 1600-fold purified enzyme with the specific activity of 65 nkat mg^-1^ as summarized in **Table 1**. However, several protein bands were still detectable on an SDS-PAGE gel (**Figure 1B**). To identify a particular protein showing the isomerase activity, the active fraction from HiTrap Blue was further separated using isoelectric focusing (**Figure 1C**). The gel slab was then sliced, separated proteins extracted and tested for the enzymatic activity. The zeatin *cis–trans* isomerase activity was found in the fraction corresponding to a 50 kDa band on the original SDS-PAGE gel (**Figure 1D**).

**Table 1:** Purification of the protein with zeatin *cis–trans*-isomerase activity from maize.

Purification step	Total activity (nkat)	Total proteins (mg)	Specific activity (nkat mg^-1^)	Purification grade (-fold)	Yield (%)
Crude extract	475	11 900	0.04	1.0	100.0
Precipitation	658	12 400	0.05	1.3	138.3
DEAE-Sepharose	724	3 110	0.23	5.8	152.2
High Q	788	1 006	0.78	19.6	165.6
Concanavalin A	162	86.20	1.89	47.1	34.1
Resource Q	145	19.00	7.66	191	30.6
Hydroxyapatite	68.1	5.00	13.6	340	14.3
HiTrap Blue HP	45.2	0.71	64.5	1 610	9.5

*Maize cobs 10 day after pollination (930 g) were used as a starting material; the activity was determined using UFLC as a production of trans-zeatin and protein content by linearized bradford method (see Materials and Methods).*

### Identification of the Purified Enzyme

MALDI-TOF/TOF MS and MS/MS allowed assigning the purified protein band with *in vitro* isomerase activity to the accession number NP_001146857 in the NCBInr database. The peptide mass fingerprinting data allowed assigning 19 peptides with *m/z* values in the range of 805–2816 Da providing a probability-based score of 131 and sequence coverage of 32.3% (Supplementary Figure S2). The MS/MS-based data provided much more convincing score value of 1345 (Supplementary Figure S3). This accession is annotated as a nucleotide pyrophosphatase/phosphodiesterase from *Zea mays* (ZmNPP; LOC100280465; Zm00001d039454_P001). The protein consists of 468 amino acids with a theoretical molecular mass of 51.2 kDa, which well corresponds to the results obtained by SDS-PAGE, and pI 6.36 determined by Compute pI/Mw (Bjellqvist et al., 1993).

### Characterization of Plant Nucleotide Pyrophosphatases/Phosphodiesterases

A BLAST analysis against translated plant genomes using ZmNPP sequence as a query returned only a few results per plant. At the time of ZmNPP identification, the database of maize genome assembly contained an additional gene with a high homology in a stretch of 160 amino acids, while the rest of the gene encoded Sir2 domain that possess a NAD-hydrolysis-dependent deacetylase activity. However, at the time of completing this paper (July 2017), the current maize genome assembly (AGPv4) contains three annotated *NPP* genes with similar length and only minor alterations (indels and several amino acid substitutions, Supplementary Figure S4). Because we were not aware of the other two ZmNPPs, we have focused in our work only on the identified enzyme. For the same reason, we refer to the enzyme as ZmNPP, without the number. Nevertheless, the mass spectrometry analysis (Supplementary Figures S2, S3) unambiguously confirmed that a protein present in the purified fraction is ZmNPP1 (Zm00001d039454_P001) and not ZmNPP2 (Zm00001d047948_P001) or ZmNPP3 (Zm00001d044311_P001). A single gene LOC_Os01g10020 was found in rice and four in *Arabidopsis*, whose genes are located on chromosome 4, next to each other (At4g29680, At4g29690, At4g29700, and At4g29710).

**FIGURE 2:**
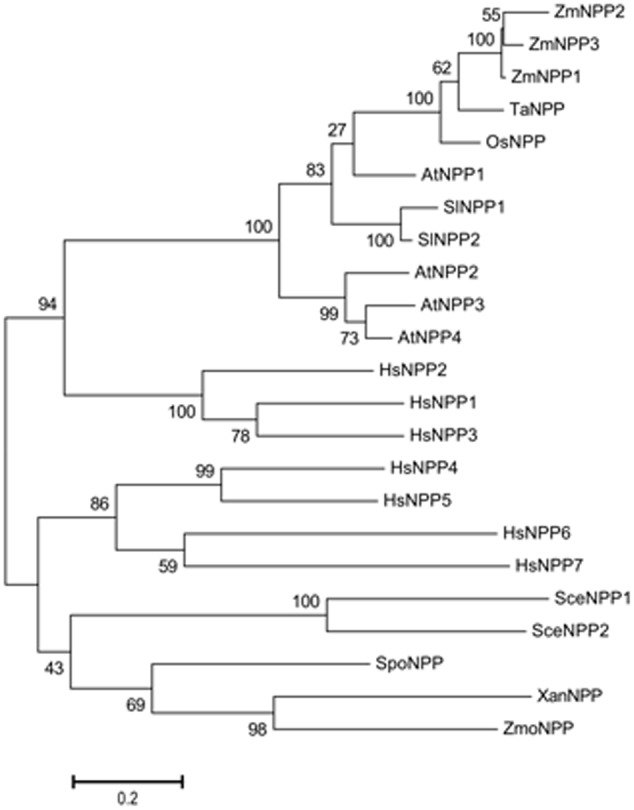
Phylogenetic analysis of nucleotide pyrophosphatases/phosphodiesterases. The following sequences were collected: *Xanthomonas* XanNPP (WP_011051855.1), *Zymomonas mobilis* ZmoNPP (WP_011241358.1), *Schizosaccharomyces pombe* SpoNPP (O94323.1), *Saccharomyces cerevisiae* SceNPP1 (P25353.2), SceNPP2 (P39997.1), *Zea mays* ZmNPP1 (Zm00001d039454_P001), ZmNPP2 (Zm00001d047948_P001), ZmNPP3 (Zm00001d044311_P001), *Oryza sativa* OsNPP (LOC_Os01g10020.1), *Arabidopsis thaliana* AtNPP1 (AT4G29680.1), AtNPP2 (AT4G29690.1), AtNPP3 (AT4G29700.1), AtNPP4 (AT4G29710.1), *Triticum aestivum* TaNPP (ADK32530.1), *Solanum lycopersicum* SlNPP1 (Solyc07g037950.1), SlNPP2 (Solyc07g037960.1), *Homo sapiens* HsNPP1 (P22413.2), HsNPP2 (Q13822.3), HsNPP3 (O14638.2), HsNPP4 (Q9Y6X5.3), HsNPP5 (Q9UJA9.1), HsNPP6 (Q6UWR7.2) and HsNPP7 (Q6UWV6.3). The sequences were aligned using CLUSTAL W method (Thompson et al., 1994). The phylogenetic tree was built by the MEGA 6.0 software by the maximum likelihood method (Tamura et al., 2013). To estimate evolutionary distance, the proportions of amino acid differences were computed using Poisson Correction Distance. The reliability of different phylogenetic clusters was evaluated by the bootstrap test (1000 bootstrap replications).

Unlike human nucleotide pyrophosphatase/phosphodie-sterase genes that contain a large number of introns and undergo an alternative splicing, all plant genes, interestingly with the exception of the additional two *ZmNPPs* (*ZmNPP2* and *ZmNPP3*), contain a single exon. As shown in **Figure 2**, plant NPP proteins cluster separately from animal enzymes in the phylogenetic tree. Mostly, when there is more than one encoding gene in the genome (e.g., in *Arabidopsis* and tomato), the paralogs are located sequentially on the same chromosome. This all suggests that before the evolutional divergence of animals and plants, there was only a single precursor gene. While there were multiple gene duplications in animals before the divergence of clades (Zimmermann et al., 2012), the genes in plants duplicated relatively recently, after the speciation.

The prediction of plant NPPs’ subcellular localization by TargetP (Emanuelsson et al., 2007) is mostly ambiguous. Two exceptions are ZmNPP2 and AtNPP3, both with high confidence to be secreted proteins. However, both of them have also predicted one trans-membrane helix by TMHMM server (Krogh et al., 2001). The other proteins’ localization is predicted with lower confidence with reliability class usually 4 or 5.

The plant NPPs in general contain all the amino acids that were shown to be crucial for the catalysis in animals (Kato et al., 2012) with two notable exceptions: Lys 277 from mouse NPP1 does not align to any specific amino acid in the plant NPPs and the Asp 308 that is crucial for substrate binding *via* water molecule is substituted with glutamate (Glu 191 in ZmNPP1) as in *Xanthomonas* enzyme. The plant enzymes are predicted to be glycosylated by NetNGlyc (Gupta and Brunak, 2002) at several asparagine residues. Without ambiguity, all enzymes shall be glycosylated at residues corresponding to Asn 108 and 208 of ZmNPP1 with the exception of AtNPP2 and AtNPP3 that have residue corresponding to Asn 108 substituted for Arg and Gly, respectively. The three *Arabidopsis* proteins that have asparagine at the position corresponding to ZmNPP1’s Lys 393 shall be glycosylated there. The last position undergoing glycosylation is the one corresponding to Asn 454 of ZmNPP1. At this position, about half of proteins are weakly predicted to be glycosylated, while the rest not to be glycosylated. Lastly, ZmNPP1, AtNPP1 and AtNPP2 shall be glycosylated at positions 35, 33 and 34, respectively. An alignment of all aforementioned plant NPP enzymes with indicated putative glycosylation sites, conserved amino acids and predicted signal peptides is shown in Supplementary Figure S4.

### Substrate Specificity of Recombinant Maize Nucleotide Pyrophosphatase/ Phosphodiesterase

To study the catalytic reaction, heterologous expression was preferred to purification from maize kernel extract for obtaining sufficient amount of ZmNPP. First attempts using *Escherichia coli* did not lead to successful protein production. Alternatively, the expression was successful using a methanol inducible expression in the methylotrophic yeast *Pichia pastoris* with the expression vector pPICZα that fuses a signal peptide to the N-terminus of the recombinant protein thus driving its secretion into the growth medium. The recombinant protein was then concentrated and purified to homogeneity (**Figure 1E**) using three relatively simple chromatographic steps as described in Section “Materials and Methods.” The size of the recombinant protein was around 60 kDa due to the presence of His-tag and a linker (3.5 kDa) and probably due to a heavier glycosylation pattern in yeast.

**FIGURE 3:**
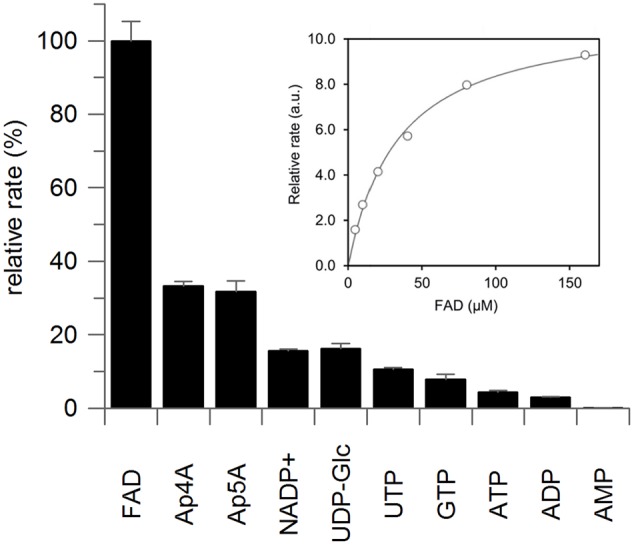
Substrate specificity of recombinant maize nucleotide pyrophosphatase/phosphodiesterase. Substrates (0.5 mM) were incubated with the purified enzyme in 100 mM McIlvaine buffer containing 20 mM MgCl_2_ at 37°C for the period of 30 min, during which both the decrease in substrate concentration and increase in the product concentration were monitored by UFLC and the tangent was used to calculate the reaction rate. Graph depicts average of three technical replicates with standard deviation. The insert shows the saturation curve of FAD hydrolysis with maximal velocity on *y*-axis expressed in arbitrary units (a.u.), from which *K*_m_ of 32.7 ± 3.0 μM was calculated by GraFit software.

The purified recombinant protein showed the zeatin *cis–trans* isomerase reaction in the presence of FAD and light. The enzyme does not significantly speed up *cis–trans* zeatin conversion in the presence of FMN; however, a non-enzymatic conversion in the presence of FMN is much faster than in the presence of FAD (Supplementary Figure S5). The obtained recombinant ZmNPP was also readily able to hydrolyze a range of typical nucleotide pyrophosphatase/phosphodiesterase substrates as shown in **Figure 3** and none of these reactions required light. With the best substrate FAD, enzyme exhibited a specific activity of 496.8 nkat mg protein^-1^ and *K*_m_ of 32.7 μM (**Figure 3**). In general, dinucleotides (FAD, diadenosine polyphosphates, NADP^+^, and UDP-Glc) were better substrates than mononucleotides. The hydrolytic reaction produced AMP from FAD, NADP^+^, ATP, and diadenosine tetra- and pentaphosphate. Further hydrolysis of produced adenosine polyphosphates was observed only after a prolonged incubation. Hydrolysis of other nucleoside triphosphates also led to monophosphate products.

### Overexpression of Maize Nucleotide Pyrophosphatase/Phosphodiesterase in Plant Systems

**FIGURE 4:**
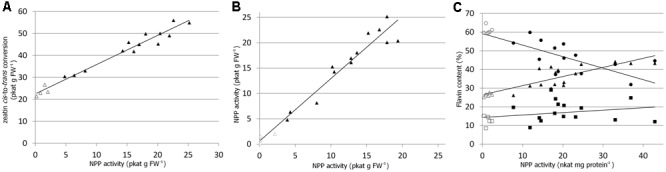
Elevated nucleotide pyrophosphatase/phosphodiesterase (NPP) activity in transgenic plants. **(A)** Nucleotide pyrophosphatase/phosphodiesterase activity assayed as the production of FMN in the extracts from various tomato hairy root lines overexpressing *ZmNPP* and correlation to zeatin *in vitro cis*-to-*trans* conversion per 1 g of fresh weight (FW). **(B)** Activity of NPP (as the production of FMN) in the presence (*y*-axis) and absence (*x*-axis) of *cis*-zeatin in various tomato hairy root lines. The reaction was set up for each sample twice identically except of the presence/absence of *cis*-zeatin. **(C)** Relation of endogenous contents of FAD (circles), FMN (triangles) and riboflavin (squares) toward the activity of nucleotide pyrophosphatase/phosphodiesterase in 4-week-old *Arabidopsis* plants overexpressing *ZmNPP*. Samples were taken from four independent lines. Open and solid symbols represent wild type and transgenic lines, respectively.

In order to quickly verify ZmNPP activity *in planta*, the *ZmNPP* gene was first expressed in a tomato hairy root system (Collier et al., 2005) and several independent transgenic lines were propagated by main root excision. The ZmNPP expressing lines showed 2–10 times higher activity of nucleotide pyrophosphatase/phosphodiesterase with FAD (assayed as the production of FMN) compared to wild type and heat inactivated samples. Similarly, zeatin *cis-*to-*trans* isomerization rate measured *in vitro* in the extracts from various transgenic lines was higher as shown in **Figure 4A**. There was a strong correlation (*R*^2^ = 0.9655) between zeatin isomerization and nucleotide pyrophosphatase/phosphodiesterase activity, which was, at the same time, independent of the presence of *cis-*zeatin (**Figure 4B**).

Further, *Arabidopsis* plants overexpressing the *ZmNPP* gene under 35S promoter were prepared. The specific activity of nucleotide pyrophosphatase/phosphodiesterase with FAD as the substrate increased in the extracts from leaves of 4-week-old *Arabidopsis* plants by an order of one magnitude compared to wild type (**Table 2**). Accordingly, the transgenic plants had decreased levels of endogenous FAD and increased levels of FMN and riboflavin (**Figure 4C**). However, no significant changes in the levels of endogenous zeatin isomers were observed. The levels of zeatins’ free bases in green and senescent leaves of *Arabidopsis* are depicted in Supplementary Figure S6. The plants also did not show any visible phenotypic changes or changes in the chlorophyll content (**Table 2**).

**Table 2:** Characteristics of *Arabidopsis* and rice plants with altered nucleotide pyrophosphatase/phosphodiesterase activity.

Plant	*Arabidopsis*				Rice				
Line	Wild type	#5	#8	#15	Wild type	#1	#2	#3	#4 (albino)
Biological replicates	5	5	4	5	9	16	3	5	3
Plants per replicate	4	4	4	4	1	1	1	1	2
Specific activity (nkat mg^-1^)	0.07 ± 0.04	1.26 ± 0.30	1.65 ± 0.61	0.86 ± 0.24	0.01 ± 0.01	0.09 ± 0.02	0.12 ± 0.02	0.05 ± 0.02	0.57 ± 0.12
*p-value*		*1.29E-03*	*0.021*	*2.53E-03*		*1.39E-10*	*4.41E-03*	*0.029*	*0.022*
Total flavins (pmol)	2.4 ± 0.5	2.3 ± 0.5	2.3 ± 0.3	2.1 ± 0.6	5.6 ± 1.5	5.8 ± 1.5	4.5 ± 1.2	6.0 ± 2.4	1.8 ± 0.2
*p-value*		*0.94*	*0.74*	*0.56*		*0.78*	*0.35*	*0.77*	*7.59E-05*
FAD (%)	61.1 ± 1.9	44.1 ± 6.0	42.5 ± 8.8	45.3 ± 8.5	51.2 ± 0.9	51.5 ± 1.5	49.5 ± 2.6	52.0 ± 1.3	55.1 ± 0.5
*p-value*		*3.20E-03*	*0.033*	*0.019*		*0.56*	*0.45*	*0.30*	*5.9E-4*
FMN (%)	26.3 ± 0.8	38.3 ± 4.1	40.3 ± 4.9	34.6 ± 5.7	46.7 ± 0.6	45.8 ± 1.3	47.7 ± 2.3	45.3 ± 1.3	34.4 ± 1.4
*p-value*		*3.57E-03*	*0.015*	*0.042*		*0.040*	*0.58*	*0.11*	*5.09E-03*
Riboflavin (%)	12.6 ± 2.3	17.6 ± 4.4	17.2 ± 5.3	20.0 ± 5.1	2.2 ± 0.5	2.7 ± 0.4	2.8 ± 0.3	2.7 ± 0.3	10.5 ± 2.0
*p-value*		*0.090*	*0.23*	*0.041*		*7.29E-03*	*0.046*	*0.063*	*0.026*
Chlorophyll *a* (μg mg^-1^)	0.71 ± 0.10	0.65 ± 0.11	0.70 ± 0.11	0.76 ± 0.12	1.09 ± 0.19	1.02 ± 0.12	1.07 ± 0.04	1.33 ± 0.24	< LOD
*p-value*		*0.45*	*0.91*	*0.46*		*0.38*	*0.80*	*0.19*	*n.d.*
Chlolophyll *b* (μg mg^-1^)	0.24 ± 0.03	0.22 ± 0.03	0.23 ± 0.04	0.25 ± 0.04	0.28 ± 0.05	0.27 ± 0.03	0.28 ± 0.02	0.37 ± 0.06	< LOD
*p-value*		*0.43*	*0.83*	*0.51*		*0.37*	*0.92*	*0.082*	*n.d.*
Chlorophyll *a*/*b*	3.01	3.00	3.05	3.04	3.85	3.86	3.84	3.59	n.d.
*p-value*		*0.73*	*0.052*	*0.13*		*0.96*	*0.93*	*0.021*	*n.d.*

*Extracts from the leaves of 4-week-old *Arabidopsis* overexpressing ZmNPP gene (LOC100280465) and 2-week-old rice plants with enhanced expression of putative nucleotide pyrophosphatase/phosphodiesterase OsNPP gene (LOC_OS01G10020) Were analyzed as described in Section “Materials and Methods.” Nucleotide pyrophosphatase/phosphodiesterase activity was measured as fad hydrolysis. results are given as mean values ± standard deviation; *p*-value was determined using student’s *t*-test against the wild type lines with number of biological replicates indicated for each line in the table; <LOD, below limit of detection; N.D., not determined.*

To assess the function of putative rice nucleotide pyrophosphatase/phosphodiesterase LOC_Os01g10020, 2-week-old rice plants in which the expression of encoding gene was enhanced by disrupting a native promoter of the gene with the promoter sequence of α-tubulin gene *OsTubA1* were examined. Originally, this only available T-DNA insertion mutant of *NPP* gene in monocot species was purchased in order to study loss-of-function phenotype; however, it later turned out that the insertion of T-DNA to the promotor region led to its overexpression rather to disruption. Approximately one fourth of the obtained plants were albinos, in which chlorophyll content was below the detection limit and the nucleotide pyrophosphatase/phosphodiesterase activity increased about 50-times compared to the wild type, while green mutant plants showed only about 10-times activity increase. The total flavin content decreased about 3-times in albinos, but remained unchanged in green mutant plants (**Table 2**).

## Discussion

In the original paper by Bassil et al. (1993), the zeatin *cis–trans* isomerase activity detected in immature seeds of common bean had following attributes: (i) the conversion required FAD or FMN, light and a reducing agent such as dithiothreitol; (ii) both zeatin isomers were substrates, although the rate of conversion of *cis-* to *trans*-zeatin was higher, and (iii) there was also a noticeable background conversion when the enzyme preparation was inactivated by boiling. Purification of a protein with the zeatin *cis–trans* isomerase activity from maize kernels led to the identification of the nucleotide pyrophosphatase/phosphodiesterase, which hydrolyzes FAD without any light requirement. NPP family of seven members has been well known in mammals. Most of human enzymes contain a single transmembrane domain residing in the plasma membrane and hydrolyze mostly nucleotides and their analogs or phospholipids (Stefan et al., 2005). Plant NPPs are less characterized. The situation is complicated by the lack of sequence determination and an overlap of the catalyzed reaction with other enzyme families, i.e., purple acid phosphatases (PAP; Li et al., 2002; Olczak et al., 2003) and Nudix hydrolases (**nu**cleoside **di**phosphate compounds linked to a moiety, X; Maruta et al., 2012), which catalyze hydrolytic breakdown of pyrophosphate and phosphodiester bonds of numerous nucleotide sugars. Chloroplast localized ADP-Glc pyrophosphatase that belongs to purple acid phosphatase family has been found to negatively regulate starch biosynthesis in rice and barley (Nanjo et al., 2006; Kaneko et al., 2014). The only plant enzyme characterized to date resembling the mammalian ones has been found in wheat (Joye et al., 2010).

The amino acid sequence of ZmNPP shows the highest similarity to the families 1 to 3 of human nucleotide pyrophosphatase/phosphodiesterases and also to the enzyme from wheat, but it lacks the transmembrane domain, which is consistent with the enzyme’s purification as a soluble protein. Correspondingly, it has been shown that some human enzymes (Belli et al., 1993; Wu et al., 2004; Sakagami et al., 2005) as well as the wheat enzyme (Joye et al., 2010) may exist in a soluble form. Accordingly to the mammalian and wheat enzymes, which are *N*-glycosylated, the ZmNPP is probably also glycosylated as predicted by NetNGlyc (Gupta and Brunak, 2002), evidenced by binding to Concanavalin A-Sepharose and a hyper-glycosylation observed on the recombinant ZmNPP protein. To determine the substrate specificity, recombinant ZmNPP was prepared in *Picchia pastoris* and purified from the culture medium. With the partially purified enzyme, the zeatin isomerization reaction proceeded even in the absence of dithiothreitol needed to inhibit the breakdown of zeatin to adenine in plant extracts that probably occurred due to the activity of cytokinin dehydrogenase (EC 1.5.99.12; Galuszka et al., 2001).

Flavins, in general, have been known to possess rich chemistry, which is exploited by nature in many enzymatic systems. However, FMN and FAD are also known to form singlet and triplet excited states upon illumination and induce non-enzymatic flavin sensitized photoisomerism of e.g., retinol, bilirubin and stilbenes by direct energy transfer (Heelis, 1982). Flavin induced photoisomerization of bilirubin is used for a treatment of neonatal jaundice (Knobloch et al., 1991). It is therefore very likely that *in vitro* ZmNPP hydrolysis of FAD to FMN and AMP induced a non-enzymatic photoisomerization of zeatin as FMN is more potent in such a process. Photoisomerization of zeatin with FAD then occurred also with the heat-inactivated enzyme, albeit in a lower rate. The conversion of zeatin isomers found *in vitro* is reminiscent of an older paper reporting on *trans-*to*-cis* isomerization of geraniol and geranyl phosphate to nerol and neryl phosphate, respectively, by cell-free extracts of carrot and peppermint, in the presence of FAD or FMN, a thiol or sulfide and light (Shine and Loomis, 1974), which may have also happened non-enzymatically by flavin sensitization.

There is not enough supporting evidence for zeatin isomerization *in vivo*. Upon feeding potato tubers with radioactively labeled *cis-*zeatin, only 5% to 9% of the label was found in *trans-*zeatin (Suttle and Banowetz, 2000). Recently, *cis–trans* isomerization was reported in pathogenic fungus *Leptosphaeria maculans* (Trdá et al., 2017). First, the authors observed an increase in *cis*- or *trans-*zeatin, when the fungus was fed with the other isomer. We have observed similar occurrence ourselves when we treated maize with micromolar concentrations of zeatins, but it was not confirmed with radiolabeled cytokinins at physiological levels (Hluska et al., 2016). Further, Trdá et al. (2017) fed the fungus with zeatins and observed accumulated isomers in the medium. Similarly to our work reported here, the *cis*-to-*trans* isomerization was approximately 3-times faster than the other way. Also, when the fungus was not added, or it was boiled prior to incubation, the conversion was smaller (Trdá et al., 2017). Thus, one can hypothesize, that nucleotide pyrophosphatase/phosphodiesterase is at play here. On the contrary, the *Arabidopsis* knock-out plants deficient in biosynthesis of either of the zeatin isomers were not able to complement its loss by isomerization (Miyawaki et al., 2006). We, therefore, prepared overexpression plants to assess whether altered nucleotide pyrophosphatase/phosphodiesterase activity affects the content or ratio of zeatin isomers *in vivo*. The FAD hydrolyzing activity in the lines expressing maize enzyme was increased 12–23-times. In these plants, FAD levels were decreased as expected. However, besides the increase in FMN, the direct product of FAD hydrolysis, part of FAD was probably converted to riboflavin. *Arabidopsis* leaves overexpressing *ZmNPP* did not show any alteration in the ratio or the total content of two zeatin isoforms. This should not be surprising considering the plants are able to suppress non-enzymatic conversion of zeatin isomers. Otherwise, the plants would have to have *trans* to *cis* ratio at the equilibrium. This may be due to several reasons, possibly because of low concentrations in the plant and limited amount of light intracellularly. Further, flavins and cytokinins are each localized to different subcellular compartments. While flavins are mainly in mitochondria and nucleus (Giancaspero et al., 2013), cytokinins are predominantly localized to apoplast (Jiskrová et al., 2016). No visible phenotype alterations were observed on these plants. Similarly, no phenotype change was observed in AtNUDX23 overexpressing plants with about 20% decrease in flavin content (Maruta et al., 2012).

To shed more light on the physiological function of plant nucleotide pyrophosphatase/phosphodiesterase, rice plants, in which the expression of *OsNPP* encoding gene was enhanced by disrupting its native promoter with a strong and constitutive promoter of α-tubulin, were examined. Approximately one fourth of the obtained plants were albinos with much higher increase in the nucleotide pyrophosphatase/phosphodiesterase activity than was observed in remaining green mutant plants. Except for a significant decrease in total flavin content in the albino plants, there was a significant shift from FMN to riboflavin. We cannot currently speculate about the lack of chlorophyll, NPP activity and flavin content, what is the cause and what is the consequence, or whether there is any causation at all. However, considering the fact we observed no albino plants of wild type origin, we can hypothesize that in some rice plants the OsNPP upregulation is so strong, it leads to severe alterations in flavin content ultimately causing chlorophyll depletion. These data are in agreement with previous observations that the reduction in the flavin content to less than 50% leads to a stunted growth and chlorosis of plants (Ouyang et al., 2010; Hedtke et al., 2011). The authors conclude, that increased photooxidative damage and down-regulated FAD-dependent cytokinin dehydrogenase, respectively are responsible for the observed phenotype.

Because of the wide substrate specificity of nucleotide pyrophosphatases/phosphodiesterases that overlaps with purple acid phosphatases and Nudix hydrolases, understanding their physiological function will require an integrative approach and research involving all these enzymes. The best understood so far is the role of purple acid phosphatase with ADP-Glc pyrophosphatase activity that diminishes the starch formation (Rodríguez-López et al., 2000; Kaneko et al., 2014). The increased expression of putative nucleotide pyrophosphatases/phosphodiesterases from *Arabidopis thaliana* after seed imbibition as can be seen in the Genevestigator database (Hruz et al., 2008) suggests they may be involved in a storage mobilization through seed germination. Very likely, the product of enzymatic hydrolysis pyrophosphate also plays a regulatory role in starch synthesis in potato tubers (Farré et al., 2000).

So far there are indices that nucleotide pyrophosphatases/ phosphodiesterases are involved in regulation of availability of redox cofactors as it has been proposed for the enzyme from *Opuntia* during fruit ripening (Spanò et al., 2011). Another role could be in pathogen defense, where the follow-up product of nucleotide pyrophosphatase/phosphodiesterase reaction riboflavin has been reported to induce a resistance against several pathogens in plants, including *Botrytis cinerea* (Azami-Sardooei et al., 2010). Indeed, the expression data for the putative nucleotide pyrophosphatase/phosphodiesterase encoded by the At4g29700 gene available from the Genevestigator database (Hruz et al., 2008) show about seven-fold increase upon *B. cinerea* inoculation. Furthermore, *Pseudomonas syringae* was shown to require guanosine tetra- and pentaphosphates for the colonization of plants (Chatnaparat et al., 2015), availability of which may be controlled by nucleotide pyrophosphatase/phosphodiesterase thus limiting the pathogen spreading.

## Conclusion

From the data presented in this work, it appears highly unlikely that nucleotide pyrophosphatase/phosphodiesterase participates in zeatin *cis–trans* isomerization *in vivo* as there is no significant change in the ratio of the zeatin isomers in plants with altered activity of this enzyme. Recently, we have postulated a hypothesis that the high levels of *cis-*zeatin type cytokinins in certain species such as maize may be a combined effect of different substrate specificity of cytokinin-specific glycosyltransferases and the ability of cytokinin dehydrogenase to preferentially degrade cytokinin *N*-glucosides (Hluska et al., 2016). While *Arabidopsis* has only a limited ability to *O-*glucosylate *cis-*zeatin (Hou et al., 2004), there are two cytokinin-specific glycosyltransferases known in maize that both prefer *cis-*zeatin to its *trans-*isomer (Veach et al., 2003). As a result, *cis-*zeatin *O-*glucosides that are resistant to the cleavage by cytokinin dehydrogenase accumulate in maize, whereas *N-*glucosides produced in *Arabidopsis* are readily degraded by the enzyme (Galuszka et al., 2007).

## Author Contributions

PG and TH designed research; TH, MŠ, and RL performed all experiments and data collection; TH, PG, and IF analyzed data; TH, IF, and PG wrote the article.

## Conflict of Interest Statement

The authors declare that the research was conducted in the absence of any commercial or financial relationships that could be construed as a potential conflict of interest.
