# Supplementary material for: Purification of Maize Nucleotide Pyrophosphatase/Phosphodiesterase Casts Doubt on the Existence of Zeatin Cis–Trans Isomerase in Plants
Source: Front Plant Sci. 2017 Aug 23;8:1473. doi: 10.3389/fpls.2017.01473 (PMC5572937; doi:10.3389/fpls.2017.01473)

Supplementary Material

Purification of maize nucleotide pyrophosphatase/phosphodiesterase casts doubt on the existence of zeatin *cis-trans* isomerase in plants

Tomáš Hluska, Marek Šebela, René Lenobel, Ivo Frébort, Petr Galuszka

Correspondence: petr.galuszka@upol.cz

Supplementary Figure S1. Typical UFLC chromatograms of enzymatic reactions. (A) shows the reaction of cis-zeatin isomerization with FAD at 0.75 min, FMN at 1.30 min, tZ at 1.55 min and cZ at 1.95 min. (B) shows typical chromatogram of FAD hydrolysis with FMN peak at 3.15 min and FAD at 3.65 min. Blue line indicates gradient of methanol, green line pressure in UFLC system, purple and black line absorbance at 268 and 449 nm, respectively.

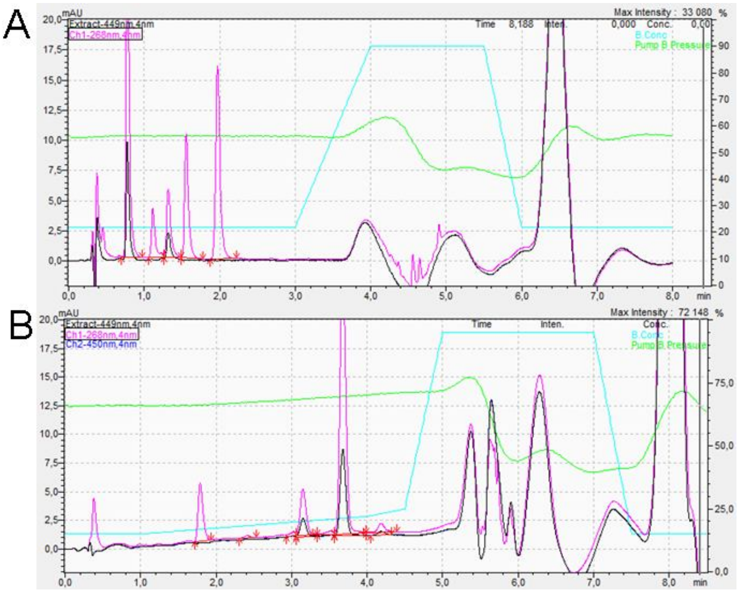

Supplementary Figure S2. Results of MALDI-TOF/TOF MS identification of protein band with zeatin *cis-trans* isomerase activity.

**Protein 1:**	LOC100280465 [Zea mays]		
**Accession:**	gi|226531542	**Score:**	131.00
**Database:**	NCBInr	**MW [kDa]:**	51.10
**Seq. Coverage [%]:**	32.30 %	**pI:**	6.39
		**No. of Peptides:**	19
**Modification(s):**	Carbamidomethyl, Oxidation		

10	20	30	40	50	60	70	80	90	100	110	120
MASPPHSVEV	RTPGDSPRPT	AALLSPSVAA	PQPSNASRLL	LLLTAAVAAA	TAFVLLRPPI	TVVTAASATA	RPLSKLSKPV	VLLISSDGFR	FGYQYKAPLP	HIRRLFANGT	SAAEGLIPVF
**130**	**140**	**150**	**160**	**170**	**180**	**190**	**200**	**210**	**220**	**230**	**240**
PTLTFPNHYS	IVTGLYPSSH	GIINNYFPDP	ISGDYFTMKN	HDPKWWLGEP	LWATAAAQGV	LSATFFWPGS	EVTKGSWNCP	DKYCRHYNGS	VPFEERVDTI	LGYFDLPPNQ	MPQFMTLYFE
**250**	**260**	**270**	**280**	**290**	**300**	**310**	**320**	**330**	**340**	**350**	**360**
DPDHQGHQVG	PDDPSITDAV	VHIDEMLGRL	IAGLEARGMF	EDVNIILVGD	HGMVGTCDRK	LVFLEELAPW	IELKSDWVLS	VTPLLAIRPP	DGVSPAEVVA	KMNEGLGSGK	VKNGEYLKMY
**370**	**380**	**390**	**400**	**410**	**420**	**430**	**440**	**450**	**460**	**470**	
LKEELPTRLH	YSESYRIPPI	IGLVGEGYKI	EMKRSKRNEC	GGAHGYDNAF	FSMRTIFAAH	GPRFQGGRTV	PSFENAEIYN	VMASILNLKP	APNNGSASFP	GTILLPNK	

m/z meas.	Δ m/z [ppm]	z	P	Range	Sequence	Modification
1630.9577	3.102	1	0	76-90	LSKPVVLLISSDGFR	
805.3849	-3.721	1	0	91-96	FGYQYK	
1589.8623	2.282	1	1	91-103	FGYQYKAPLPHIR	
803.4890	0.4263	1	0	97-103	APLPHIR	
1442.5965	1.727	1	1	195-205	GSWNCPDKYCR	Carbamidomethyl: 5, 10
842.5132	4.535	1	0	270-277	LIAGLEAR	
1828.0556	-3.451	1	1	300-314	KLVFLEELAPWIELK	
2816.5555	-0.07056	1	0	315-341	SDWVLSVTPLLAIRPPDGVSPAEVVAK	
1279.6700	-1.122	1	1	359-368	MYLKEELPTR	
1295.6715	3.933	1	1	359-368	MYLKEELPTR	Oxidation: 1
1054.4965	1.202	1	0	369-376	LHYSESYR	
2391.2725	0.7624	1	1	369-389	LHYSESYRIPPIIGLVGEGYK	
1355.7942	0.6609	1	0	377-389	IPPIIGLVGEGYK	
1857.0556	0.1071	1	1	377-393	IPPIIGLVGEGYKIEMK	
1873.0493	-0.5566	1	1	377-393	IPPIIGLVGEGYKIEMK	Oxidation: 16
2088.8738	-1.189	1	1	397-414	RNECGGAHGYDNAFFSMR	Carbamidomethyl: 4
1932.7761	0.4818	1	0	398-414	NECGGAHGYDNAFFSMR	Carbamidomethyl: 3

m/z meas.	Δ m/z [ppm]	z	P	Range	Sequence	Modification
1948.7639	-3.170	1	0	398-414	NECGGAHGYDNAFFSMR	Carbamidomethyl: 3; Oxidation: 16
969.5245	-1.992	1	0	415-423	TIFAAHGPR	

Supplementary Figure S3. Results of MALDI-TOF-TOF MS/MS identification of protein band with zeatin *cis-trans* isomerase activity.

**Protein 1:**	LOC100280465 [Zea mays]		
**Accession:**	gi|226531542	**Score:**	1345.46
**Database:**	NCBInr	**MW [kDa]:**	51.10
**Seq. Coverage [%]:**	29.10 %	**pI:**	6.39
		**No. of Peptides:**	19
**Modification(s):**	Carbamidomethyl, Oxidation		

10	20	30	40	50	60	70	80	90	100	110	120
MASPPHSVEV	RTPGDSPRPT	AALLSPSVAA	PQPSNASRLL	LLLTAAVAAA	TAFVLLRPPI	TVVTAASATA	RPLSKLSKPV	VLLISSDGFR	FGYQYKAPLP	HIRRLFANGT	SAAEGLIPVF
**130**	**140**	**150**	**160**	**170**	**180**	**190**	**200**	**210**	**220**	**230**	**240**
PTLTFPNHYS	IVTGLYPSSH	GIINNYFPDP	ISGDYFTMKN	HDPKWWLGEP	LWATAAAQGV	LSATFFWPGS	EVTKGSWNCP	DKYCRHYNGS	VPFEERVDTI	LGYFDLPPNQ	MPQFMTLYFE
**250**	**260**	**270**	**280**	**290**	**300**	**310**	**320**	**330**	**340**	**350**	**360**
DPDHQGHQVG	PDDPSITDAV	VHIDEMLGRL	IAGLEARGMF	EDVNIILVGD	HGMVGTCDRK	LVFLEELAPW	IELKSDWVLS	VTPLLAIRPP	DGVSPAEVVA	KMNEGLGSGK	VKNGEYLKMY
**370**	**380**	**390**	**400**	**410**	**420**	**430**	**440**	**450**	**460**	**470**	
LKEELPTRLH	YSESYRIPPI	IGLVGEGYKI	EMKRSKRNEC	GGAHGYDNAF	FSMRTIFAAH	GPRFQGGRTV	PSFENAEIYN	VMASILNLKP	APNNGSASFP	GTILLPNK	

Cmpd.	No. of Cmpds.	m/z meas.	Δ m/z [ppm]	z	Rt [min]	Score	P	Range	Sequence	Modification
	1	1759.0511	1.99	1		130.9	0	75-90	S.KLSKPVVLLISSDGFR.F	
	1	1630.9552	1.57	1		131.8	0	76-90	K.LSKPVVLLISSDGFR.F	
	1	1302.7463	3.59	1		37.6	0	79-90	K.PVVLLISSDGFR.F	
	1	1589.8526	-3.87	1		23.3	0	91-103	R.FGYQYKAPLPHIR.R	
	1	803.4888	0.19	1		43.7	0	97-103	K.APLPHIR.R	
	1	1442.5953	0.92	1		57.6	0	195-205	K.GSWNCPDKYCR.H	Carbamidomethyl: 5, 10
	1	842.5075	-2.30	1		58.0	0	270-277	R.LIAGLEAR.G	
	1	2816.5538	-0.66	1		112.1	0	315-341	K.SDWVLSVTPLLAIRPPDGVSPAE VVAK.M	
	1	1718.9823	1.36	1		73.0	0	325-341	L.LAIRPPDGVSPAEVVAK.M	
	1	1279.6730	1.17	1		66.8	0	359-368	K.MYLKEELPTR.L	
	1	1295.6672	0.60	1		51.0	0	359-368	K.MYLKEELPTR.L	Oxidation: 1
	1	1054.4941	-1.05	1		68.9	0	369-376	R.LHYSESYR.I	

Cmpd.	No. of Cmpds.	m/z meas.	Δ m/z [ppm]	z	Rt [min]	Score	P	Range	Sequence	Modification
	1	1355.7946	0.94	1		90.9	0	377-389	R.IPPIIGLVGEGYK.I	
	1	1873.0452	-2.73	1		37.7	0	377-393	R.IPPIIGLVGEGYKIEMK.R	Oxidation: 16
	1	1857.0562	0.43	1		74.5	0	377-393	R.IPPIIGLVGEGYKIEMK.R	
	1	1932.7772	1.05	1		109.7	0	398-414	R.NECGGAHGYDNAFFSMR.T	Carbamidomethyl: 3
	1	1948.7717	0.80	1		45.7	0	398-414	R.NECGGAHGYDNAFFSMR.T	Carbamidomethyl: 3; Oxidation: 16
	1	1472.6410	2.31	1		46.2	0	402-414	G.GAHGYDNAFFSMR.T	
	1	969.5270	0.52	1		70.2	0	415-423	R.TIFAAHGPR.F	

Supplementary Figure S4. Alignment of plant nucleotide pyrophosphate/phosphodiesterase protein sequences. Identical and similar amino acids are shaded in black and gray, respectively. Amino acids important for substrate binding are marked with asterisk (numbering as in ZmNPP1): Phe 125, Asn 145, Trp 187 (part of WPG motif), Glu 191 (that is homologus to Asp 308 in mouse NPP1), Tyr 207 and Asp 243. Asparagine residues predicted to be glycosylated are marked with #: Asn 108, Asn 208, Asn 454 and asparagine in AtNPP2 to 4 corresponding to Lys 393; asparagine residues 35, 33 and 34 in ZmNPP1, AtNPP1 and AtNPP2 are not marked. Predicted signal peptides giving a reliable prediction are shaded with dark blue.

```
                    10         20         30         40         50         60         70         80
            ....|....| ....|....| ....|....| ....|....| ....|....| ....|....| ....|....| ....|....|
ZmNPP1    1 -------MAS PPHSVEVRTP GDSPRPTAAL LS----PSVA APQP------ -SNASRLLLL LTAAVAAATA FVLLR-PPIT
ZmNPP2    1 -------MAS PPHSVEP--- ---------- ---------- ---------- -SIASCLLLL LTAAVAAATA FVLLR-LPIT
ZmNPP3    1 -------MAS PPHSFEVRTP GDSPRPTAAL LS----PSVA APQP------ -STASRLLLL LTAAVAAATA FVLLH-LPTT
TaNPP     1 -------MAA PPISGHPHSS VDSPLPTDAL LAHPQLPSAP APQA------ -PTASRFLII LTAALAVSTS YLLLLRPPFS
OsNPP     1 -------MAA AAAAAPPFAA GDSPPPTALL LPRTTTTTAG AAPAPR---R SSASSRLHLL LTAALAVATS YLLLI-LPRT
AtNPP1    1 MISGDTLSAK KPKSVPQEED -QDPPSQSIA LLDNHTDSSG SDSSTRSISS CFIFTSLLLV TCIALSAASA FAFLFFSSQK
AtNPP2    1 -------MTK SKPGRSGFSG ---------- ---------- ---------- -YILYKLSLT VLIVLSVAVT AN----GSDS
AtNPP3    1 -------MAK TKNVIS-FKL ---------- ---------- ---------- -SLIFLLNIF IVATIAAAAA VNAGTKGLDS
AtNPP4    1 ---------- ---------- ---------- ---------- ---------- ---------- ---------- ----------
SlNPP1    1 -MNSDSISFK -PMAVPTRED DEEPPISSTS LLSFDTDDCS TSIPPKPPFS SLILTPLILV TCVSLSTAII FAYLFFS-HS
SlNPP2    1 -MNSNSVSFK -PMAVLTKED -EDPPISSTS LLSFDTDDCS PSSPTKTPFN SVILTALILV TCVSLSTAVT FAFLFFS-HS

                    90        100        110        120        130        140        150        160
            ....|....| ....|....| ....|....| ....|....| ....|....| ....|....| ....|....| ....|....|
ZmNPP1   62 VVTAASAT-- --------AR PLSKLSKPVV LLISSDGFRF GYQYKAPLPH IRRLFANGTS AAEGLIPVFP TLTFPNHYSI
ZmNPP2   39 VVTAASAT-- --------AR PLS---KPVV LLISSDGFCF GYQYKAPLPH IHRIFANGTS ATEGLVPVFP TLTFPNHYSI
ZmNPP3   62 VVTAASAT-- --------AR PLSKLSKPVV LLISSDGVRF GYQYKAPLLH IRHLFANGTS AAEGLVPVFP TLTFPNHYSI
TaNPP    67 AVSAAASTSS F-------AR PLSKLPKPVV LLISSDGFRF GYHHKAPTPH IRRLIANGTS AAEGLIPVFP TLTFPNHYSI
OsNPP    70 PLSAAPAPAA A-------AR AQVKLEKPVV ILISSDGFRF GYQHKAATPH IHRLIGNGTS AATGLVPIFP TLTFPNHYSI
AtNPP1   80 PVLSLNQISK SPAFDRSVAR PLKKLDKPVV LLISSDGFRF GYQFKTKLPS IHRLIANGTE AETGLIPVFP TLTFPNHYSI
AtNPP2   39 PSSYVRRP-- ---------Q PPKKLNKPVV LLISCDGFRF GYQFKTETPN IDLLISRGTE AKTGLIPVFP TMTFPNHYSI
AtNPP3   42 RPSKTRRP-- ---------W PFKKLNKPVV LMISCDGFRF GYQFKTDTPN IDLLISGGTE AKHGLIPVFP TMTFPNHYSI
AtNPP4    1 ---------- ---------- ---------- ---------- ---------- ---------- ---------- ----------
SlNPP1   78 SVSSISHVSR ---------- PLQKLKHPVV LLISSDGFRF GYQYKTDTPN IRRLITNGTE AELGLIPVFP TLTFPNHYAI
SlNPP2   77 SISSISHVTR S--------R PLQKLKHPVV LLISSDGFRF GYQYKTDTPN IRRLITNGTE AELGLIPVFP TLTFPNHYAI
                                                                         #                  *

                   170        180        190        200        210        220        230        240
            ....|....| ....|....| ....|....| ....|....| ....|....| ....|....| ....|....| ....|....|
ZmNPP1  132 VTGLYPSSHG IINNYFPDPI SGDYFTMKNH DPKWWLGEPL WATAAAQGVL SATFFWPGSE VTKGSWNCPD KYCRH-YNGS
ZmNPP2  106 VTGLYPSSHD IINNYFPDPI SGDYFTMKNH DPKWWLGEPL WATAAAQGVL SATFFWSGSE VTKGSWNCPD KYCRH-YNGS
ZmNPP3  132 VTDLYPSSHG IINNYFPDPI SGDYFTMKNH DPKWWLVEPL WATAAAQGVL SATFFWPGSE VTKGSWNFPD K---------
TaNPP   140 VTGLHPSSHG IINNFFPDPI SGDNFNMGSH EPKWWLGEPL WVTAADQGVQ ASTFFWPGSE VKKGSWDCPD KYCRH-YNGS
OsNPP   143 ATGLYPSSHG IINNYFPDPI SGDYFTMSSH EPKWWLGEPL WVTAADQGIQ AATYFWPGSE VKKGSWDCPD KYCRH-YNGS
AtNPP1  160 VTGLYPAYHG IINNHFVDPE TGNVFTMASH EPEWWLGEPL WETVVNQGLK AATYFWPGSE VHKGSWNCPQ GLCQN-YNGS
AtNPP2  108 ATGLYPASHG IIMNKFTDPV SGELFNRN-L NPKWWLGEPL WVTAVNQGLM AATYFWPGAD VHKGSWNCPK GFCKAPYNVS
AtNPP3  111 ATGLYPAYHG IIMNKFTDPV TGEVFNKG-L QPKWWLGEPL WVTAVNQGLK AVTYFWPGSE VLKSSWTCPE GYCPH-FNLS
AtNPP4    1 ---------- ---------- ---------- ---------- ---------- ---------- ---------- ----------
SlNPP1  148 VTGLYPAYHG IINNYFLDPN IREPFTMASY DPKWWLGEPL WETVVNHGLK AATYFWPGSE VNKGGWTCPE YFCKI-YNGS
SlNPP2  149 VTGLYPAYHG IINNNFLDPI SGEHFTMGSH DPKWWLGEPL WETVVNHGLK AATYFWPGSE VNKGGWTCPE SLCKR-YNGS
                          *                                             *   *                  *#
```

```
                250        260        270        280        290        300        310        320
           ....|....| ....|....| ....|....| ....|....| ....|....| ....|....| ....|....| ....|....|
ZmNPP1 211 VPFEERVDTI LGYFDLPPNQ MPQFMTLYFE DPDHQGHQVG PDDPSITDAV VHIDEMLGRL IAGLEARGMF EDVNIILVGD
ZmNPP2 185 VPFEER---- ---------- ---------- ---------- ---------- ---------- -----ARGMF EDVNIILVGD
ZmNPP3 202 ---------- ---------- ---------- -----GHQVG PDDPSITDAV VHIDEMLGRL IAGLEARGMF EDVNIILVGD
TaNPP  219 VPFEDRVDAV LGYFDLPVDE MPQFLTLYFE DPDHQGHQVG PDDPAITDAV THIDEMIGRL IAGLEARGMF EDVNIILVGD
OsNPP  222 VPFEERVDAI LGYFDLPSDE MPQFLTLYFE DPDHQGHQVG PDDPAITEAV VRIDEMIGRL IAGLEERGVF EDVNIILVGD
AtNPP1 239 VPFDDRVDTI LSYFDLPSNE IPSFMTLYFE DPDHQGHQVG PDDPQITEAV VNIDRLIGRL IDGLEKRGIF EDVNVIMVGD
AtNPP2 187 VPLEERVDTI LNYFDLPERE IPDFMALYFD EPDIQGHEYG PDDPRVTEAV SKVDKMIGRI IMGLEKRKVF SDVHVILLGD
AtNPP3 189 VPLEERVDSV LSHFDHLEDE VPDLLMLYFD EPDQSGHNYG PDDPRVTTAV SRVDKMIGRV IKGLKQREIF DEVHVILLGD
AtNPP4 1   ---------- ---------- ---------- ---------- ---------- ---------- ---------- ----------
SlNPP1 227 VPFEERVDTV LNYFDLPNDE IPSFMTLYFG DPDHQGHKVG PDDPQITEAI ARVDGMIGKL IQGLEERGVF EDVNIIMLGD
SlNPP2 228 VPFEERVDTV LKYFDLPNDE IPSFMTLYFE DPDHQGHKVG PDDPQITEAI ARVDSMIGKL IKGLEARGVF EDVNIIMVGD
                                                *

                330        340        350        360        370        380        390        400
           ....|....| ....|....| ....|....| ....|....| ....|....| ....|....| ....|....| ....|....|
ZmNPP1 291 HGMVGT--CD RKLVFLEELA PWIELKSDWV LSVTPLLAIR PPDGVSP--- ----AEVVAK MNEGLGSGKV KNGEYLKMYL
ZmNPP2 206 HGMVGT--CD RKLVFLEELA PWIELKSDWV LLMTPLLAIR SPDGVSP--- ----TEIVAK MNEGLGSGKV KNGEYLKMYL
ZmNPP3 248 HGMVGT--CD RKLVFLEELA PWIELKSDWV LSMTPLLAIR PPDGVSP--- ----DEVVAK MNEGLGSGKV KNGEYLKMYL
TaNPP  299 HGMVGT--CD QKLLFLEELA PWIEVKADWV LSRTPLLAIR PPDGISP--- ----SEVVAK MNEGLSSGKV KNGQYLKMYL
OsNPP  302 HGMVGT--CD KKLVFLDELA PWIKLEEDWV LSMTPLLAIR PPDDMSL--- ----PDVVAK MNEGLGSGKV ENGEYLRMYL
AtNPP1 319 HGMVGT--CD KKLVVLDDLA PWIKIPSSWV QYYTPLLAIQ PPSGHDA--- ----ADIVAK INEGLSSGKV ENGKYLKVYL
AtNPP2 267 HGMVTN--CD KKVIYIDDLA DWIKIPADWI QDYSPVLAMN PRWGKDVKNP GQKNAELVRK MNEALSSGKV ANGEFLQVYL
AtNPP3 269 HGMVTNCECN EKAIYIDDLA DWIKIPAAWI QAYSPVLAIN PQWGKDVENQ SEKNAEVVAK MNEALSSGKV KNGEFLKVYL
AtNPP4 1   ---------- ---------- ---------- ---------- ---------- ---------- MNEALSSGKV KNGEFLTVYL
SlNPP1 307 HGMVGT--CD KKLIFLEDLA RWIKIPKDWI QSYSPLLSIR PPRSYSA--- ----KDVVTK MNEGLKSGKV KNGQNLKVYL
SlNPP2 308 HGMVGT--CD RKLIFLEDLA PWIDIPKDWI QSYSPLLSIR PPPSYSA--- ----KDVVTK MNEGLKSEKV KNGQYLKVYL

                410        420        430        440        450        460        470        480
           ....|....| ....|....| ....|....| ....|....| ....|....| ....|....| ....|....| ....|....|
ZmNPP1 362 KEELPTRLHY SESYRIPPII GLVGEGYKIE MKRSKRNECG GAHGYDNAFF SMRTIFAAHG PRFQGGRTVP SFENAEIYNV
ZmNPP2 277 KEELPTRLHY SESYRIPPII GLVGEGYKIE MKRSKRNECG GAHGYDNAFF SMRTIFVAHG PRFQGGRTVP SFENVEIYNV
ZmNPP3 319 KEELPTCLHY SESYRIPPII GLIGEGYKIE MKRSKRNECG GAHGYDNAFF SMRTIFATHG PRFSGW---- ----------
TaNPP  370 KEDLPSRLHY SESYRIPPII GLVAEGYKVE MKVSKKNECG GAHGYDNAFF SMRTIFIAHG PRFEEGKIVP SFVNVEIYNA
OsNPP  373 KEDLPSRLHY ADSYRIPPII GLPEEGYKVE MKRSDKNECG GAHGYDNAFF SMRTIFIAHG PRFEGGRVVP SFENVEIYNV
AtNPP1 390 KEDLPSRLHY VDSDRIPPII GLVDEGFKVE QKKSKAKECG GAHGYDNAFF SMRTIFIGHG PMFSKGRKVP SFENVQIYNV
AtNPP2 345 KENLPQRLHY SDSSRIPPII GMVGEGLMVK QNRTYVQECS GTHGYDNMFF SMRSIFVGYG PRFRRGIKVP SFENVQVYNA
AtNPP3 349 KEKLPERLHF SESYRIPPII GIVGEGLMVR QNRTNAQVCY GDHGYDNELF SMRTIFVGHG SRFSRGKKVP SFENVQIYNV
AtNPP4 21  KEKLPDRLHY SQSYRIPPII GMVGEGLIVR QNRTNAQECY GDHGYDNKFF SMRTIFVGHG SRFRRGKKVP SFENVQIYSV
SlNPP1 378 KEELPDRLHY SESDRIPPII GLIDEAFKVE QKSSKRFECG GSHGYDNAFL SMRSIFIGHG PKFARGRKVS SFENVQIYNM
SlNPP2 379 KEELPDRLHY SASDRIPTII GLIDEAFKVE QKSSKRFECG GAHGYDNAFF SMRTIFIGHG PKFASGRKVP SFENVQIYNV
                                                 #

                490        500        510
           ....|....| ....|....| ....|....| ...
ZmNPP1 442 MASILNLKPA PNNGSASFPG TILLPNK--- ---
ZmNPP2 357 MASILNLKPT PNNGSASFPG TIPLPNK--- ---
ZmNPP3 384 ---------- ---------- ---------- ---
TaNPP  450 IASILSLKPA PNNGSSSFPE TVLLPSE--- ---
OsNPP  453 IASILNLEPA PNNGSSSFPD TILLPSE--- ---
AtNPP1 470 ISSILGLKAA PNNGSDEFSS SILLPRV--- ---
AtNPP2 425 VAEILGLRPA PNNGSSLFTR SLLLPFGETS QVE
AtNPP3 429 VAELLGLRPA PNNGSSLFPR SILLPSGATR EVK
AtNPP4 101 VADILGLRPA PNNGSSLFPR SILLPFRATR GLE
SlNPP1 458 VTTILNIQGA SNNGTSSFPK TILLPSH--- ---
SlNPP2 459 VTKILNIQGA PNNGTTTFPD TILLPSH--- ---
                      #
```

Supplementary Figure S5. Comparison of *cis*-to-*trans* conversion of zeatin isomers (0.2 mM) in a presence of FAD and FMN. Reactions were set up with or without purified recombinant ZmNPP enzyme and incubated in 100 mM McIlvaine buffer containing 20 mM $MgCl_2$, 0.1 mM FAD/FMN, at 37°C under white fluorescent light or in the dark for 20 minutes; determined by UFLC; graph depicts average of three technical replicates with standard deviation.

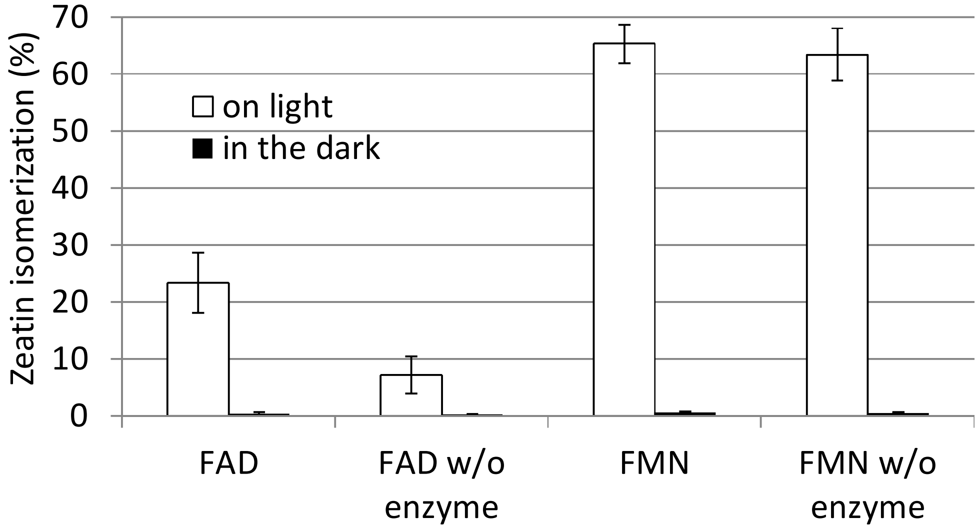

Supplementary Figure S6. Level of *trans*-zeatin and *cis*-zeatin in green (A) and senescent (B) leaves of Arabidopsis of wild type or expressing ZmNPP. None of the results was significantly different (Student's t-test; $p < 0.05$; $n = 4$) between the wild type and ZmNPP plants.

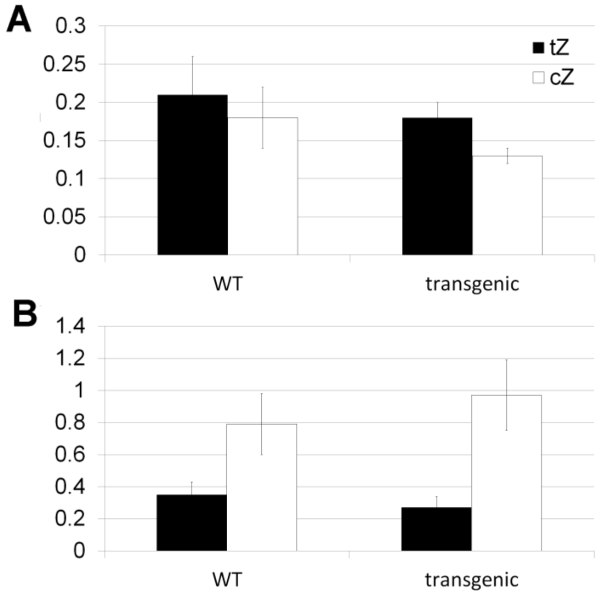

Supplement: Supplementary file 1 [file Presentation_1.pdf]
